# The Dentist of the Future

**Published:** 1873-07

**Authors:** 


### DENTAL REGISTER.

Vol. XXVII.] JULY, 1873. [No. 7.

### THE DENTIST OF THE FUTURE.

*Read by W. P. Morrill, before the Joint Session of Indiana and Kentucky State Dental Associations, June 5th.* 1873.

"American Dentistry, from the earliest annals, must be conceded to be of significance, interest and profit. Casting our eyes over that period when dentistry first began its existence, we recall the giants, not pigmies, who lived in those days. Among these were Gardette, Greenwood, Harris, Parmleys, Townsend, Badger, Maynard, and many others, who become fixed stars in this firmament of pioneer dentistry, and whose effulgence still glows with a steady ineffaceable radiance. We venerate their names and their deeds, for they were glorious. Conspicuous as these bright examples are in our history, conspicuous as many illustrious names which still live to adorn our profession to-day, we cannot resist the temptation of throwing forward the horoscope, and of snatching the gleam of what shall be *the dentist of the future.*

Comparatively unknown to the pioneers of dental practice were the researches of science and the ingenuity of art now made subservient to our purposes. The precious germs of knowledge, once so scarce, have multiplied and been spread broadcast in our day, so that each representative of our vocation may increase his store of knowledge without stint or hindrance. Nothwithstanding these sunny avenues of our intellectual life, sweet and attractive as the palm colonnades of a tropical clime, where the fountains of truth scatter their gems, the number of dental practitioners who wander in these grateful retreats, and pause to strip the waters of science, are alas, too few. We could wish that responsibilities of the future, which is to be a period not of apathy but one of industry and science, could be more evenly distributed.

Defective education, formidable and wide spread, besets our progress ; but the echoes of Reform ! Reform ! reverberate from the shores of Atlantic to the slopes of the Sierras along the Pacific. The exigencies of the times, and requirements of humanity, alike demand that he who would reach the *ultima thule* of our profession must possess high attainments. Broader and more thorough culture is demanded. A preliminary education, high and liberal, is an indispensable requisite, as much so as in other professions. The curricula of dental colleges are tending toward improvement and excellence. The training and discipline necessary for a candidate to receive the honors of these valued institutions, and to fit him to enter an honorable carreer of dental practice, must be painstaking and scrupulously exact. It will be no disparagement to our illustrious prototypes who have adorned our profession, nor to those who still live to grace it with becoming honors, to say that the dental practitioner of the future must possess greater qualifications than his predecessors. Much broader and more catholic principles, and more unselfish tenets prevail to-day among dentists than at any time previous in our history. Prejudice and petty bickering among the cultivated as well as among unlearned of our calling ; while men freer and more enlightened in their moral nature are encouraging social and friendly intercourse. The free offerings of our collective counsels and experience which these associations themselves impart, like the irrepressible air and light of heaven, infuse fresh energy into the monotonous existence of every practitioner. These organizations, binding together with adamantine chains professional and fraternal sympathy, are the instrumentalities which are destined to encompass the whole land with their recuperative energy. Assurances such as these are written upon the lintels of our profession. We are then not left in doubt as to what shall be the qualifications of him who shell be *the dentist of the future.*

The sphere of scientific research expands daily. An acquaintance with the minute and exact discoveries of medical science should be the laudable ambition of every practitioner of dentistry who expects large results. There is no one with a brilliant record here to-day who does not in a large degree possess this culture. These observations, made richer and more valuable by experience as time rolls on, will become the inheritance of successors. Natural and more rational modes of treatment will then be understood and practiced. The diagnosis will be accurate, penetrative and discovering. The utility and value of the teeth in their relations to human economy, to say nothing of beauty, will be better appreciated even among dentists. Conservative methods for the teeth's preservation will be more persistent and rewarded with higher triumphs. Three-fourths of the cases we see deformed with hideous artificial dentures under this new guidance and superior skill will be wearing their own natural teeth. Extraction of teeth—knights of the forceps—will be among the lost arts. Labor-saving machines and mechanical appliances beyond anything we know of, will continue to be brought to still higher perfections and greater utility. These facilities will improve the character and increase the permanence of work performed. Our text books, embracing the results of scientific discoveries, will furnish valuable assistance to the student of the future, containing as they will, well digested principles of dental science. In the list of subjects treated of in the standard works will be: dental physiology, dental pathology, dental microscopy and anatomy, dental therapeutics, dental chemistry, dental surgery and operations by a score of authors. It must be confessed with no little mortification that but few works of this kind at present adorn any of our libraries. All the advantages to be derived from this service so indispensable to our present needs, will be reserved for our more fortunate successors. The current periodical literature of the profession now so fragmentary, loose and ephemeral, will have more solid and enduring character. Those who shall have charge of this department hereafter will be men who can devote all their time and attention to this one pursuit.

In taking part in these triumphs of profession the student will have claim to rank fellowship, and recognition among the learned. In the domain of art, which is an indispensable adjunct to the specialty of dentistry, the results reaching higher ideals in expression, and continuing the assimulative and fusing processes of the imagination—methods far beyond any now known will be the temporary field of the future votaries of our calling. It is useless to predict what is fast becoming an accomplished fact; that of making the manufacture of artificial dentures a separate department. Even now there are numerous dental practitioners who devote themselves entirely to operative dentistry, lopping off the mechanical branch so far as dentures are concerned. This indicates the exercise and concentration of all the powers and faculties in one given direction, thus increasing the efficiency of this department.

With all these civilizing forces and world moving tendencies urging us to duty and to truth, we can neither remain idle nor listless spectators but earnest co-workers to carry forward the general interests of dentistry which are to have such an important bearing in the future.

The great want of the present, as auxiliary to this preparation, is the tone of public sentiment. If we had some way not degrading, by which we could communicate hygienic laws to the masses; make them acquainted with the nature and value of dental operations, then would charlatanism have few votaries and honest merits receive greater rewards. We might, I think, lay aside some of the dignity of our calling, invade even the confines of ethics, without being inconsistent with correct notions of propriety, and have this instruction given, even though the channels of the press be employed. The clergymen do not rely upon supernatural powers alone to keep the truths of the gospel and revelation alive, but arouse the people to a due respect for the lessons they teach. The conservation of the teeth may not have that eternal importance, but during our sojourn on earth it is some comfort to know that we possess knowledge and skill adequate to preserve these organs in natural lifetime ; and a confidence in this information with which to inspire the masse to a proper tone, would materially lessen the burdens of our profession. Time in office practice does not allow us to give this instruction. How every dentist knows the comfort and ease he experiences the moment a patient approaches accustomed to dental operations ; but if we have ignorance and skepticism to baffle with and fritter away our time, how laborious and unsatisfactory will be our services.

I have now indicated, imperfectly it is true, the preliminary ground work which time and experience assure me is in reserve for *the aentist of the future.* These requirements are far beyond any period in our history. That they are already responded to with alacrity by no inconsiderable number of our profession, is true. The ability, strength, and faith which the masses are also beginning to repose in our calling, is a more hopeful promise carrying encouragement with it. Anticipating the day when judicious instruction in the laws of life will more generally prevail, when mental, moral, and physical obligation shall be corrected, giving to us a nobler manhood, then will our profession certainly have less discomforts than those we experience to-day. The precise period can not be foretold, but not in remote future will the character and influence of the dental professsion be more sensibly felt among the masses. Punctuality in attendance of patients on their engagements should be more prompt. In the success of the practitioner of the future people will take more pride will esteem it their bounden duty, to pay the dentist regular visits, as among the important affairs of life. He will become their true counsellor and guide. The scope of his opinions will embrace not merely present needs of the case, but extend from etiology to an assured prognosis. Public sentiment thus elevated will not hesitate between the meritorious and the pretender. Empiricism will be left without dupes. Our State laws for the protection of the people against quackery will be useless.

These prospects now seeming Utopian for the dentist of the future, I have abiding faith to believe are not over drawn but imperfectly sketched. Nevertheless, as a co-worker upon the living tissues of that mystic life to which our profession is mainly dedicated, I bid you not in burning words of Napoleonic grandeur that "forty centuries look down upon you," but from the heights of the past and present ages, "countless worthies of our godlike profession point and beckon to a goal more elevated than ever attracted legislators and conjurors, Solons and Cæsars."

